# Advances and opportunities in integrating economic and environmental performance of renewable products

**DOI:** 10.1186/s13068-022-02239-2

**Published:** 2022-12-22

**Authors:** Samir Meramo, Peter Fantke, Sumesh Sukumara

**Affiliations:** 1 The Novo Nordisk Foundation Center for Biosustainability, Technical University of Denmark, Kemitorvet 220, 2800 Kgs. Lyngby, Denmark; 2 Quantitative Sustainability Assessment, Department of Environmental and Resource Engineering, Technical University of Denmark, Produktionstorvet 424, 2800 Kgs. Lyngby, Denmark

**Keywords:** Bio-based products, Life cycle, Biotechnology, Sustainability assessment, Technology readiness levels, Bioprocess optimization

## Abstract

There is a growing global need to transition from a fossil-based to a bio-based economy to produce fuels, chemicals, food, and materials. In the specific context of industrial biotechnology, a successful transition toward a sustainable development requires not only steering investment toward a bioeconomy, but also responsibly introducing bio-based products with lower footprints and competitive market prices. A comprehensive sustainability assessment framework applied along various research stages to guide bio-based product development is urgently needed but currently missing. To support holistic approaches to strengthen the global bioeconomy, the present study discusses methodologies and provides perspectives on the successful integration of economic and environmental performance aspects to guide product innovation in biotechnology. Efforts on quantifying the economic and environmental performance of bio-based products are analyzed to highlight recent trends, challenges, and opportunities. We critically analyze methods to integrate Techno-Economic Assessment (TEA) and Life Cycle Assessment (LCA) as example tools that can be used to broaden the scope of assessing biotechnology systems performance. We highlight the lack of social assessment aspects in existing frameworks. Data need for jointly applying TEA and LCA of succinic acid as example commodity chemical are assessed at various Technology readiness levels (TRLs) to illustrate the relevance of the level of integration and show the benefits of the use of combined assessments. The analysis confirms that the implementation of integrated TEA and LCA at lower TRLs will provide more freedom to improve bio-based product’s sustainability performance. Consequently, optimizing the system across TRLs will guide sustainability-driven innovation in new biotechnologies transforming renewable feedstock into valuable bio-based products.

**Supplementary Information:**

The online version contains supplementary material available at 10.1186/s13068-022-02239-2.

## Background

The recent success in scaling up bio-based innovations [1] has created expectations of decreasing the current dependence on fossil-based materials in a transition toward a circular economy and renewable energy systems [2]. Bioeconomy envisions to embody activities required to shift into resource-efficient and carbon-neutral economies worldwide [3]. This economy concept involves prioritizing opportunities to produce renewable bio-resources and their transformation, including waste streams, into bio-products, such as fermented food, biochemicals, biomaterials, and bioenergy [4]. The demand for bio-based products has seen progressive growth in the past decade [5]. The biochemicals, in their entirety, comprised a 2% share of the global chemical sector by 2008; projections indicate that the trend is expected to rise to 25% by 2025 [6]. The production of bio-based products has the potential to be more environmentally sustainable than fossil-based products. Therefore, shifting from a finite fossil resource-based economies to a renewable and circular bio-based economy might boost transitioning toward a sustainable development [7]. Most biochemicals have potential benefits compared with their fossil-based counterparts, e.g., lower greenhouse gas (GHG) emissions [8–10]. However, if overlooked, bio-based products could perform worse in others impact categories, such as land use, water consumption, among others [11]. Thus, claiming sustainability of bio-based products requires a better understanding of the actual environmental impacts and options for minimizing those as function of the specific bio-based production system at hand. From a global perspective, chemical pollution has exceeded its planetary boundary [12]. Galan-Martin et al. [13] examined the implications of shifting to a renewable and bio-based chemical industry, finding that the alternatives with highest GHG emissions savings exceed the planetary boundary for biodiversity loss by 30%. Rockström et al. [14] defined this boundary as the rate of biodiversity loss measured in species extinction rate, as extinctions per million species-years (E/MSY), proposing a boundary of 10 E/MSY. Further studies must include planetary boundaries to guarantee a successful transition into a sustainable bioeconomy [14].

Alongside the existing issues regarding the environmental sustainability performance of bio-based production, the scaling up costs of their transformations prevails as a significant challenge to remain competitive. Performing relevant research and development (R&D) across various life cycle stages provides insights for product optimization and boost commercialization efforts [15]. The administration and R&D encircling a biotechnology innovation have to be of the highest quality to attract attention from the venture capitalist community [16]. If any of these became exceedingly missing, biotechnology innovation would be effectually worthless and jeopardize commercialization levels.

There is an absence of clarity to recognize and prioritize critical issues that could affect the sustainability performance of bio-based products. Identifying current strengths, weaknesses, opportunities, and threats (SWOT) toward sustainability in the bio-based economy might boost the vision for enhancing innovations to develop technologies, products, or services [17]. Such lucidity can only be achieved if the research is sustainability-driven, in line with the current bioeconomy goals [1] and technological development phases [18]. Sustainability assessment can aid the identification of SWOT, while the technology takes steps forward toward commercialization. The level of maturity is commonly described by the Technology readiness levels (TRLs) (1 to 9), which identify and rank R&D stages and level of development of a product, process, or technology [19, 20]. Then, innovations undergo R&D phases while progressing across TRLs and nearing production, scale-up, and commercialization, eventually. A persisting challenge in biotechnology development is the low rate of successful commercialization of innovations, where less than 1% of innovations derived from academia currently reaches industrial practice globally [21]. Due to the challenges in translating the lab-scale success to pilot-scale, as the TRLs increase, several ventures fail and the phenomenon of such downfall is often referred as the Valley of Death [22]. Hence, to survive the valley of death stakeholders need to identify, and avoid the factors (i.e., through early sustainability assessments and identification of SWOT) which may lead to the failures, early in the development stages (TRLs 1–3) and through the process (TRLs 4–7) [23] until the innovation is matured enough to match commercial expectations (TRLs 8–9).

Bio-based platforms are fundamentally supported by renewable feedstocks, comprising products derived from first-generation biomass, primarily at high TRLs [24]. Important market drivers focus on offering environmentally friendlier and more renewable solutions to substitute petrochemicals [25]. Bio-based products from second-generation biomass are currently under development, at low-to-mid-TLRs. Lower quantities of readily accessible sugars and high pretreatment costs have hindered the translation of R&D activities for second-generation biomass toward higher TRLs [26]. Novel processes to valorize alternative renewable feedstocks with low to mid TRLs are investigated. These include macro-algae [27], micro-algae [28], and municipal wastes residues [29], often referred to as third- and fourth-generation biomass [24]. A few years ago, the European Commission (EC) provided a comprehensive overview of the development needs and status for the sugar-based platform [30]. The state of many biochemicals is listed based on the TRLs. The selection criteria for the literature review carried out in this work include related publications on those bio-based products from the EC list that might be relevant due to their maturity or applications. The emphasis of the work presented here does not cover studies on biofuels (e.g., bioethanol, biodiesel, etc.) that serve the energy sector but includes those bio-based products with potential applications in food, chemical, materials, and other industries.

To ensure that bio-based products are not only environmentally sustainable but also viable, economic aspects need to be considered in addition [31]. The Techno-economic Assessment (TEA) is a widely used tool for assessing technical and economic issues of a production plant or manufacturing system [32]. TEA and Life Cycle Assessment (LCA) serve as starting points for integrating economic and environmental sustainability performance in biotechnology R&D. Comprehensive decision-making requires identifying conflicting trade-offs, which are not entirely settled if the analysis focuses on separate TEA and LCA [33]. It is urgent to explore ways of integrating economic and environmental performance based on sustainability-driven objectives for boosting innovations. Many integrated LCA and TEA studies have been conducted on bio-based products as discussed elsewhere [33–35]. Recently, integration efforts were analyzed from a chemical process design perspective [34]. Most investigations have focused on separate TEA and LCA studies, while proper integration remains an ongoing task. In addition, the relevance of the TRLs in biotechnology product development from a life cycle perspective needs to be further explored. Previous studies have addressed the need of including TRL in the early sustainability assessment [36, 37] to promptly investigate the existing tradeoffs. As a result, the manuscript focuses on analyzing the efforts to integrate TEA and LCA with application in the bio-based production and the relevance of TRLs in the assessment task. The early quantification of environmental and economic sustainability performance will enhance the identification of SWOT and avoid the factors that lead to the Valley of Death. With this in mind, we present an overview of current ways to combine or integrate economic and environmental sustainability performance in biotechnology R&D. In addition, challenges to operationalize TEA and LCA integration are outlined. A case study on succinic acid production is applied, illustrating the challenges and calling for a combined sustainability assessment to guide the R&D phases across TRLs. Finally, ways forward to optimize systems are discussed to minimize trade-offs and successfully boost sustainability-driven innovation of renewable bio-based products.

## TEA and LCA implementation in guiding R&D activities in bio-based production

In recent years, inclusion of TEA and LCA in R&D stages in biotechnology has surfaced to evaluate benchmarks and find hotspots independently [35]. Techno-economic aspects are assessed during the technology development to steer economic and technical innovations [38]. This methodology determines research priorities by identifying cost bottlenecks during the early design stages [39]. TEA enables closed-loop feedback that connects economics, process modeling, and laboratory data in a continuous stepwise improvement scheme to progress over the TRLs [40]. LCA quantifies the environmental performance of a product, process, or service during its life cycle [41]. Contrary to TEA, fewer LCA studies have been applied to bio-based products [42, 43]. These studies are mostly limited to gate-to-gate [44] or cradle-to-gate [45] assessments. Other contributions have used the LCA to benchmark upstream and downstream technologies [46]. Still, LCA studies are absent for many widely traded biochemicals, such as fumaric acid or methyl levulinate [30], which remain at low levels of maturity (< 5 TRLs). Table 1 summarizes some major LCA studies (the list is not exhaustive) applied to bio-based products based on the report given by [30], classifying them by TRLs, biomass source, and assessed system boundaries. It is worth highlighting that the purpose was to show that at least one LCA study was conducted for each of the listed biochemical.

**Table 1:** LCA studies of bio-based building blocks and their corresponding TRLs

TRLs	Compound	Feedstock	Processing pathway	System boundaries	Refs.
8–9	Acetic acid	Poplar chips (2nd generation)	Fermentation	Cradle-to-gate	[52]
	Lactic acid	Glucose (1st generation)	Hydrolysis	Cradle-to-grave	[53]
	1,3-Propanediol	Glucose (1st generation)	Fermentation	Cradle-to-gate	[54]
	1,4-Butanediol	Wheat straw (1st generation)	Direct fermentation	Cradle-to-gate	[55, 56]
	Ethylene	Poplar chips (2nd generation)	Rectisol reaction	Cradle-to-gate	[57]
	Propylene glycol	Oils (1st generation)	Esterification	Cradle-to-gate	[58]
	Ethylene glycol	Miscanthus (2nd generation)	Catalytic conversion	Cradle-to-gate	[59]
	Furfural	Sugar beet (1st generation)	Pyrolysis	Gate-to-gate	[60]
	Sorbitol	Glucose (1st generation)	Hydrogenation	Cradle-to-gate	[61]
7–8	Succinic acid	Sorghum (1st generation)	Fermentation	Cradle-to-gate	[62]
6–7	Levulinic acid	Rice straw (2nd generation)	Acid dehydration	Cradle-to-gate	[63]
	Ethyl lactate	Corn stover (2nd generation)	Reversible esterification	Cradle-to-grave	[64]
5–6	Butyric acid	Wheat straw (2nd generation)	Fermentation	Cradle-to-gate	[65]
	Formic acid	Beechwood (2nd generation)	OxFA-Process	Gate-to-gate	[66]
	5-Hydroxymethyl furfural	Bread waste (2nd generation)	Catalyst conversion	Cradle-to-gate	[67]
	2,5-Furandicarboxylic acid	Hardwood chips (2nd generation)	Catalyst reaction	Cradle-to-gate	[68]
4–5	Adipic acid	Forest residues (2nd generation)	Fermentation	Cradle-to-gate	[69]

### Integration of TEA and LCA

**Fig. 1:**
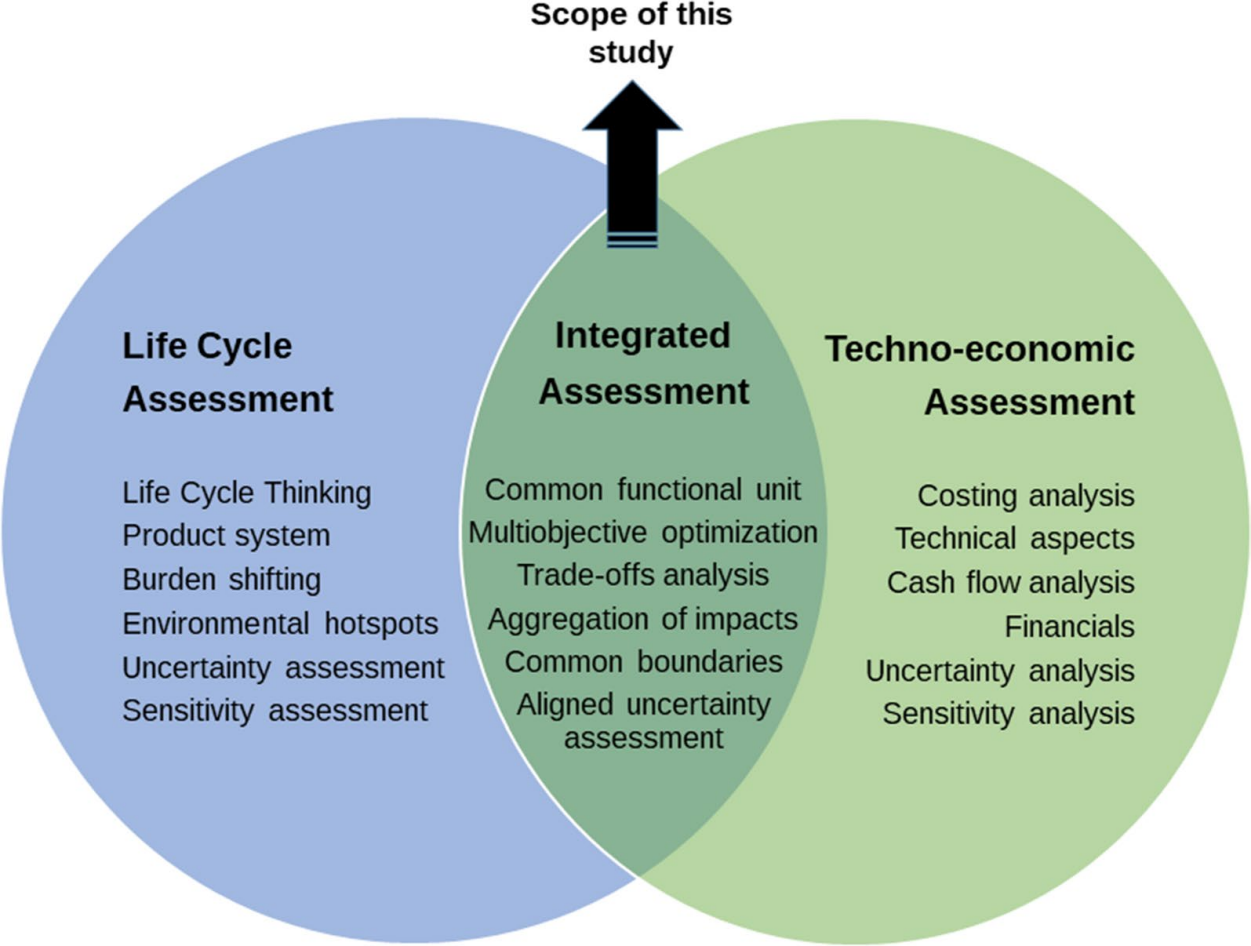
Venn diagram highlighting the differences between LCA, TEA, and integrated assessments. Focus on studies addressing integration methods

Integration approaches for combining TEA and LCA already exist, each with their specific limitations. Analyzing the trade-off derived from the economic and environmental performance evaluation is an essential task to guide sustainability-driven innovation. However, the purpose of this integration is not fully achieved if TEA and LCA are performed independently and inconsistently. Consistent integration of these methodologies involves systematically combining financial, technical, and environmental performance aspects to provide deep insights on the trade-offs that guides researchers in the technology development. Figure 1 shows a common set of goals and scope that the TEA and LCA needs to attain for seamless and consistent integration.

Several studies in the literature have addressed TEA and LCA of bio-based products, including assessments of lignocellulosic biorefineries [47], co-production of lactic acid and ethanol [48], or biohydrogen and biomethane production [49]. Those studies that do not apply any integration (i.e., common functional unit, aligned boundary conditions, multiobjective optimization, or monetization factors) are not considered in the search.

### Technology maturity and freedom of design in biotechnology R&D

**Fig. 2:**
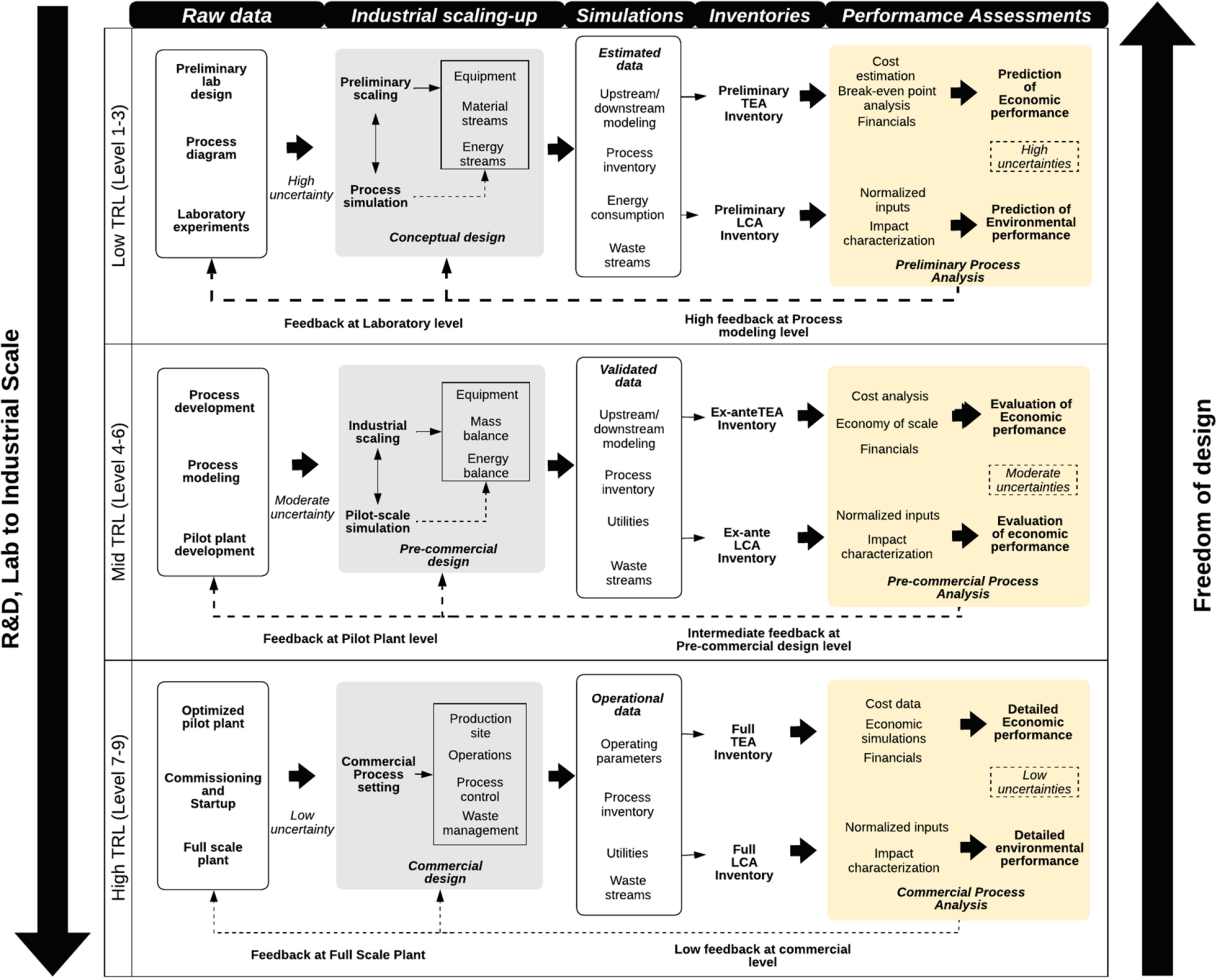
R&D stages for sustainable innovation in biotechnology in developing sustainability assessment across TRLs. Assessment methodology within the TRLs (left-to-right), freedom of design related to Technology development (top-to-bottom)

In addition, the level of technology’s maturity is an essential aspect in technology development and sustainability assessment of biotechnological innovations. The lower the TRL, the more freedom of design exists at the cost of higher uncertainties. Moderate levels of both uncertainties and design freedom are expected for intermediate TRLs. Figure 2 displays a schematic representation of technology assessments for R&D tasks across TRLs.

To initiate the iterative assessment, one begins from available data to perform preliminary LCA and TEA to obtain an elevated level of feedback to optimize the technology. Ex-ante LCA and TEA inventories are generated at this stage, determining economic and environmental performance [50]. At higher TRLs, lower design freedom and uncertainties are observed, but contrastingly a rigorous LCA and TEA could be performed. Sustainability assessment is not often practiced in the initial stages delaying, in some cases, the sustainable development across the TRLs. Few combined TEA–LCA studies (in bio-based production) have assessed technologies at low maturity levels, implying that most assessments were performed at higher TRLs.

Trade-offs exist when applying strategies for improving environmental and economic performance [51]. Once the TEA and LCA are integrated, such trade-offs can be observed and iterated with the R&D activities to optimize bioprocesses. Hence, it is critical to invest in methodological efforts to develop integrated TEA and LCA frameworks. Implementing such comprehensive methodologies to screen promising biotechnology innovations, rank research priorities, and map the path for R&D is critical for bringing sustainable bio-based products into the market. Therefore, the contribution describes an overview of the efforts and challenges in integrating the methods of LCA and TEA, and the evaluation of a case study to show the associated trade-offs. The efforts are essential for the primary goal of this work, to provide directions for future research toward a consistent and successful integration of economic and environmental performance in biotechnological innovations.

## Overview of TEA and LCA integration efforts

Sustainability assessment identifies trade-offs and combines the economic and environmental performance of a product system. Integrating TEA and LCA will directly contribute to tuning the process and optimizing bio-based production systems. Strategies and methods in process design [70], process synthesis [71], and process integration [72] (e.g., design of water recycling networks) have addressed some applicability of combined approaches [73]. However, the efforts for integrating TEA and LCA are geared toward the assessment phase. Stand-alone assessment is a widespread practice to evaluate sustainability as this helps to determine scaling-up settings, environmental profiles, and economic performance of a bio-based production [74]. Separate, standalone LCA and TEA can identify potential improvement areas [35] and also help estimate carbon credits and offsets at the process or supply chain levels [75].

Nevertheless, additional multicriteria decision-making would provide more comprehensive assessment platforms [76]. Stand-alone LCA and TEA studies typically fail to uncover relevant trade-offs across environmental and economic aspects [34, 77, 78]. R&D technology phases based on TRLs need to be streamlined in combined approaches, considering quantitative (and qualitative) strategies across TRLs [79]. The main goals of combining TEA and LCA can vary substantially. Identified objectives include:

- Determining system hotspots and comparing the outcomes with a standard reference (e.g., well-established process or technology).
- Evaluating project alternatives for process design, feedstock, or product applications.
- Comparing technologies using generic methods to set targets and benchmarks.

There is no standard approach for combining TEA and LCA. Efforts made toward an integrated approach have employed quantitative strategies. A common characteristic of the reported combined methodologies is the exclusion of the linkages between the economic and environmental performance outcomes, which also includes separately considered sensitivity and uncertainty aspects. The above is also extended to the application of sensitivity and uncertainty analyses independently. Table 2 describes key characteristics of proposed TEA and LCA integration strategies at different integration levels and system boundaries. Literature provides a broad range of different integration methodologies which have been applied at various extents. These methodologies include multicriteria decision analysis (MCDA), multiobjective optimization (MOO), the Life Cycle Sustainability Assessment (LCSA), monetization methods, among others. The methodologies are classified into three main groups:

- Contributions that introduce methods to assess combined economic and environmental indicators.
- Assessment methods that are used for conducting optimization when multiple decision objectives are required by stakeholders. Among these methods, we find MCDA, MOO, and others are listed.
- Contributions that encompass the effect of change in input variables, resulting in variations in environmental and economic performance, and introducing sensitivity approaches to foresee impacts of such changes.

**Table 2:** Key aspects of efforts made to integrate TEA and LCA considering integration framework and system boundaries

Framework	Method/approach	Scope	Key characteristics of proposed methods	Refs.
Combined indicators	GHG abatement cost	Life cycle (cradle-to-grave)	Straightforward application for a wide range of systems Appropriate for studies based on CO_2_ reduction Requires setting performance targets	[80, 104]
	Monetization of impacts	Life cycle (cradle-to-grave)	Straightforward application for a wide range of systems Appropriate for optimization-based studies Requires defining monetization factors	[78]
	Normalized impacts	Life cycle (cradle-to-grave)	Measures the relativity performance of economic and environmental indicators Applies linear interval standardization for normalization	[105]
	Sustainability footprint	Partial Life cycle (cradle-to-gate or gate-to-gate)	The method is based on statistical analysis Focuses on technologies comparisons and optimization Requires the assignation of weighting factors	[81]
	GREENSCOPE^1^	Partial Life cycle (gate-to-gate)	Multiple indicators for several areas Visualization in spider/radial diagrams Requires setting performance targets	[82]
	Sustainability assessment by impact normalization	Partial Life cycle (gate-to-gate)	Impact normalization using GREENSCOPE approach Application for coal gasification technologies The focus is on climate change and energy impacts	[106]
	Aggregation metrics based on ROI^2^	Partial Life cycle(gate-to-gate)	A metric for the extension of a financial metric Adequate for process optimization and integration Requires the assignation of weighting factors	[83, 84, 95]
	MOO combined with superstructure optimization	Partial Life Cycle (gate-to-gate)	Application in process synthesis and analysis Performance measured by Net Present value and Global Warming potential Trade-offs visualization in a 2D-Pareto	[87]
	MOO based on trade-off analysis	Partial Life cycle (gate-to-gate)	Analyses the trade-offs of CO_2_ emissions and net profit Integrates simulations and sustainability assessment Deals with a multiobjective optimization	[88]
	MOO based on ROA and stochastic analysis	Partial Life Cycle (well-to-wheel)	Both deterministic and stochastic analysis are implemented Integration based on an MOO including ROA analysis	[89]
MCDA or MOO	MCDA based on Stakeholders analysis	Life cycle (cradle-to-grave)	Includes a stakeholder's analysis and social indicators Trade-off visualization in a 2D-Pareto Rates indicators in terms of relevance, practicality, reliability, and importance	[90]
	MCDA based on TOPSIS^3^	Partial Life cycle (gate-to-gate)	Applies aggregation based on positive and negative ideal solutions (best and worst targets) The geometric distance is used to normalize indicators Requires the assignation of weighting factors	[91]
	Hybrid Sustainability assessment and process integration	Partial Life cycle (cradle-to-gate)	Combines sustainability assessment with process integration and experimental validation Employs the analytical hierarchy process for MCDA Material and energy streams are represented using arrays	[92]
	MCDA for biorefinery supply chain under uncertainties	Partial Life cycle (cradle-to-gate)	Deals multiobjective optimization with stochastic modeling Market uncertainties are considered Trade-off visualization in a 2D-Pareto	[94]
	Thermo-economic optimization	Partial Life cycle (gate-to-gate)	Focuses on technologies comparison and CO_2_ reduction Combines energy integration with economic and environmental assessment Trade-off visualization in a 2D-Pareto	[96]
	Uncertainty of combined performance	Partial Life cycle (gate-to-gate)	Application for biorefinery design and optimization Employs Monte Carlo simulation Uncertain inputs are set via Spearman's rank correlation coefficients	[97]
Linkages of TEA and LCA results	Sensitivity of economic penalties of CO_2_ avoidance	Life cycle (cradle-to-grave)	Includes an MCDA combined with interpretation phase Focuses on the strategic planning level Evaluates sensitivity between economic penalty for CO_2_ avoidance and energy consumption	[98]
	Life cycle optimization	Life cycle (cradle-to-gate)	Focuses on supply chain optimization Trade-off visualization in a 2D-Pareto Sensitivity analysis evaluates the influence of product selling price on environmental and economic performance	[99]
	Weighting-based optimization	Life cycle (cradle-to-gate)	Sensitivity evaluates the effects of weighting factors in the overall performance optimization function Monte Carlo analysis is included Non-linear optimization based on a weighted objective function	[100]
	Life cycle sustainability assessment	Partial Life cycle (gate-to-gate)	Integrates environmental, economic, and social sustainability Sensitivity analysis evaluates overall performance for each sustainability dimension	[102]
	Fuzzy and Monte Carlo optimization	Partial Life cycle (gate-to-gate)	Combines Fuzzy theory and Monte Carlo simulation Employs a set of indicators to measure sustainability Requires the assignation of weighting factors	[103]

^1^Gauging reaction effectiveness for the environmental sustainability of chemistries with a multiobjective process evaluator

^2^Sustainability weighted return on investment metric

^3^The technique for order of preference by similarity to an ideal solution

### Combined indicators for sustainability quantification

A first attempt of combined economic and environmental indicators included cost reduction by effects of improving the environmental performance. Verma et al. [80] estimated decarbonization or life cycle greenhouse gas (GHG) emissions reduction-related costs based on a normalization factor for a project to a reference target. Applying similar approaches is foreseeable, considering the efforts to reduce global emissions and economic penalties, such as carbon-based taxes. Consequently, finding optimal options based on minimized carbon abatement costs at process design and supply chain levels delivers significant advantages to management and policy makers. A TEA–LCA monetization-based method was presented by Ögmundarson et al. [78]. The method defines a common functional unit (e.g., 1 kg of bio-based product) and generates environmental and economic output data. Monetization of environmental impacts aggregates TEA and LCA results and shows a single score per functional unit that is easy to understand for stakeholders.

The selection, evaluation, and optimization of indicators can make a system incrementally more sustainable. Selecting key performance indicators (KPI) applicable to all systems is not straightforward. In bioprocesses, the complexities are shared between different systems. Material and energy sources, hazardous materials, exposure from chemicals, water systems, wastes, land use, cost, safety, and occupational aspects are amongst the parameters that embody such constraints in this industry from a life cycle perspective. The task is completed by collecting performance indicators in augmented metrics. The industry has adopted a few of them addressing the described sustainability areas.

Sikdar et al. [81] described statistical features of proposed sustainability metrics, considering less than ten indicators, addressing the sustainability footprint concept based on Euclidean and geometric means. Ruiz-Mercado et al. [82] presented a taxonomy of sustainability indicators within The Gauging Reaction Effectiveness for the Environmental Sustainability of Chemistries with a Multiobjective Process Evaluator (GREENSCOPE) approach focused on gate-to-gate assessments. More than 140 indicators were listed, embracing various categories (environment, material efficiency, energy efficiency, and economics). El-Halwagi [83] introduced an indicator-based method for aggregating sustainability indexes to prioritize and assess process improvement initiatives. The metric extends the conventional economic return on investment to rank the favorable or detrimental effects on sustainability performance at the early design stage. Other scholars have proposed related augmented metrics to embrace additional aspects such as process safety [84] or exergy [85] focused on the chemical process design level.

### Optimization-based approaches for sustainability quantification

Multicriteria approaches have played a relevant role in integrated assessments as alternative aggregation methods. MCDA and MOO methods introduced in the previous section is used to rank systems to combine environmental and economic outcomes and choose the optimal alternative [86]. Optimization algorithms are implemented to identify the viable solution space based on the enforced constraints. Data visualization and decision-making analysis in MOO commonly include the display of the set of non-dominated solutions (Pareto front) [87]. The non-dominated solutions are common problems in multiobjective optimization, as the result of the restriction of improving one objective function without simultaneously decreasing the performance at least one of the other objectives [86]. In the context of sustainability assessments, Pareto curves serve to analyze systems under an integrated perspective, in which CO_2_ emissions can be measured, while the algorithm maximizes the net profit [88]. Kern et al. [89] implemented the real options analysis (ROA) to combine economic and environmental performance along with MOO and stochastic modeling. Halog and Manik [90] presented an MCDA framework adopting a life cycle approach. The method included a stakeholder analysis and dynamic system modeling. A Pareto displays sustainability criteria, indicators, and hotspots through agent-based modeling, data envelopment analysis, and sustainability network theory. Optimization approaches are strategic at the early design stages, where feedback-feedforward loops combined with TEA and LCA have shown successful results [88]. MCDA also deals with laboratory-scale designs using TEA and estimates environmental parameters, such as carbon footprint. LCA module gives the overall impact characterization. Then, gathered data are combined through a decision-making framework [91]. Other optimization approaches have aggregated more process design modules combining analytical hierarchy process, TEA, LCA, and process integration [92]. The Economic Input–Output Life-Cycle is a combined optimization framework based on mass and energy balance data [93]. The LCA uses matrix arrangements to represent inlet/output data and a scaling vector to scale the technology. The following stages implicate economic evaluation, risk analysis, and process integration. Decision-making frameworks are relevant to support sustainability quantification. Detailed economic and environmental modeling and multiobjective optimization have shown promising outcomes to assess biorefinery supply chains [94]. Some studies have used hybrid optimization and combined indicator approaches [94–96].

### Environmental and economic variations and sensitivity approaches

Contributions focused on analyzing the linkages between economic and environmental objectives considering sensitivity and uncertainty analyses have traditionally analyzed under separate perspectives. Lactic acid production was assessed by TEA and LCA under uncertainties with Monte Carlo simulations and Latin hypercube sampling. Sensitivities of the product selling price, carbon footprint, and energy consumption were obtained [97]. MCDA approaches were undoubtedly found to have the most in-depth understanding of the linkages between environmental and economic outcomes. The sensitivity of economic penalties of CO_2_ reduction is of high interest for strategic planning and environmental policy [98]. Analyzing the linkages of TEA and LCA outcomes is critical at the supply chain level. Objective functions can deal with economic and environmental targets constrained by transportation, bioprocessing, upgrading, and others [99]. The Pareto displays the optimal solution space, quickly identifying the trade-offs between the two objectives. The evaluation of uncertainties and sensitivities combined with MCDA and MOO approaches might lead to more complicated methodologies. Analyzing environmental and economic outcomes through such approaches has involved combined indicators and non-linear programming [100]. Ways of decreasing their complexity include weighting optimization functions [101], evaluation several scenarios [102], and Monte Carlo simulations [103]. Graphical representations significantly help describe the bonds between the economic and environmental results [77].

## Challenges of integrating LCA and TEA for sustainability quantification

LCA studies on bio-based monomers, materials, or chemicals have seldom included all life cycle stages, e.g., final product usage, recycling, and waste disposal [52, 61, 78]. Even though such evaluation simplifies their complexity, such simplifications might lead to biased results and inaccurate conclusions, often due to burden-shifting [107, 108]. Many studies have faced difficulties in expanding the TEA boundaries [77, 109], most linked with gate-to-gate approaches [110]. These assessments help inform stakeholders of bioprocessing level outcomes competing with the equivalent product(s). For systems with similar upstream data (e.g., same supply chain for a common feedstock), gate-to-gate assessments tend to be similar in scope to cradle-to-gate assessments. Full LCA is remarkably complex and challenging to be generalized into a computational-based formulation [111].

### LCA and TEA harmonization and consistency issues

The integration of LCA and TEA requires harmonization, considering raw data and the scope/goal assessment phase [112]. This relates to the lack of consistency in defining criteria and methodological aspects for integrating environmental and techno-economic aspects. A unique integrated solution cannot sufficiently serve all drivers and the technology’s R&D activities. Ögmundarson et al. [78] explained that setting accurate (non-subjective) values to monetization factors is a key challenge. Unexpected variations in these factors might appear for specific environmental indicators considering different macro- and microeconomic features. This condition leads to dealing with high uncertainty levels in the assigned values. In addition, the interpretation phase is not fully covered or analyzed in many contributions. How to communicate the results and their linkages remains challenging. Besides, the difficulty in applying TEA into a life cycle thinking implicates that this methodology does not follow a standard, as LCA does.

### Methodology selection and integration approaches

Many of the reported integration efforts have included indicator-based methods [78, 82, 85] and optimization approaches [87, 113] based on sustainable design. Sustainability embraces not only environmental and economic dimensions, but also includes a societal dimension [114]. However, in most cases, the social sustainability performance has been entirely or partially dismissed in the integrated methodologies [115]. The quantification of the social impact performance of production systems is undoubtedly a challenging task [116], and more research is needed to overcome this limitation. Another essential aspect of developing and applying integrated sustainability assessment is the selection of appropriate methodologies that guarantee the consistency of the combined assessment.

Azapagic et al. [117] emphasized the relevance of suitable indicator selection on early stage process design if the combined evaluation must inform various stakeholders with contradictory interests. A variety of combined indicators has been proposed to characterize impact categories per value-added. This approach aims to evaluate all life cycle stages, but they are still limited to gate-to-gate boundaries. Santoyo-Castelazo and Azapagic [118] introduced a multi-objective decision-support framework under MCDA combined with indicator-based data collection. The visualization of results is included by displaying indicator performances via spider diagrams [119]. A significant drawback of this approach is the difficulty of being comprehensible for a broad audience when the set of indicators is large, or the number of evaluated alternatives is high.

The LCSA was presented by Finkbeiner et al. [120], describing a comprehensive framework considering Life Cycle Costing (LCC), traditional LCA, and social assessment. The LCSA presents a rational approach that combines, reads, and transfers knowledge from different sustainability dimensions [102]. Similarly, to include the social dimension into the decision support, the Social Life Cycle Assessment (SLCA) was introduced by Hoogmartens et al. [121]. The SLCA methodology assesses potential social effects of a product, process, or service in its life cycle [116]. The evaluated aspects include but are not limited to human rights, working conditions, health and safety, among others. Both LCSA and SLCA dismissed the relevance of considering the expectations of stakeholders as a decisive factor in the assessment focusing more on LCA and LCC. In addition, these methodologies require additional data from other assessments. Recently, Hauschild et al. [122] discussed the combination of risk assessment (for safety) and LCA (for sustainability) in the context of safe and sustainable-by-design decisions to support the development of new technologies and products. Comprehensive methodologies are needed, since complex decisions might lead to practitioners’ misunderstandings. This condition will persist if the system shows trade-offs for conflicting decisions. As mentioned, methodology selection is still challenging for practitioners; therefore, developing frameworks to support their selection becomes crucial.

### Model representativeness and technology maturity

The optimization-based integration approaches are not exempt from challenges. Chen and Grossmann [123] explained that MCDA and MOO deal with Pareto-curves that display a set of optimal (non-inferior) scenarios over the solution space. Optimal decisions must be performed regularly, considering trade-offs between conflicting objectives [124]. Searching for non-inferior solutions in intrinsic nonconvex problems adds another level of difficulty. Dealing with these systems is still challenging, not exclusively associated with MOO or MCDA in quantitative sustainability assessment but multiple fields [125, 126]. As an alternative, multicriteria analysis studies have included analytical hierarchy process approaches to assign scores and rank criteria weights [127].

Traditionally, TEA has been used in R&D phases to provide feedback in loop-based procedures, technology maturity relates to data quality and associated uncertainties during this assessment. In addition, LCA has been applied in technology development and as design support tool [128, 129], similarly as TEA. Thus, identifying TRLs of the examined technologies is incredibly advantageous when determining what integration methodology can be applied [19]. Not many studies have included the technology maturity concept, more associated with terms, such as ‘emerging’ [130] or ‘immature’ [77]. There are missing methods that characterize the R&D phases by a standardized system, such as the TRLs. This framework is broadly accepted in the scientific community and widely used in the chemical industry and scientific platforms, such as the Horizon 2020 program [131]. New methods must integrate this conception to better assimilate the technical, economic, and environmental aspects that result in the early identification of SWOT. This would boost the progress of current methodologies (and overcome current limitations) to the next level, emphasizing the early optimization of production systems.

## A case study to highlight trade-offs and integration challenges in bio-based production

**Fig. 3:**
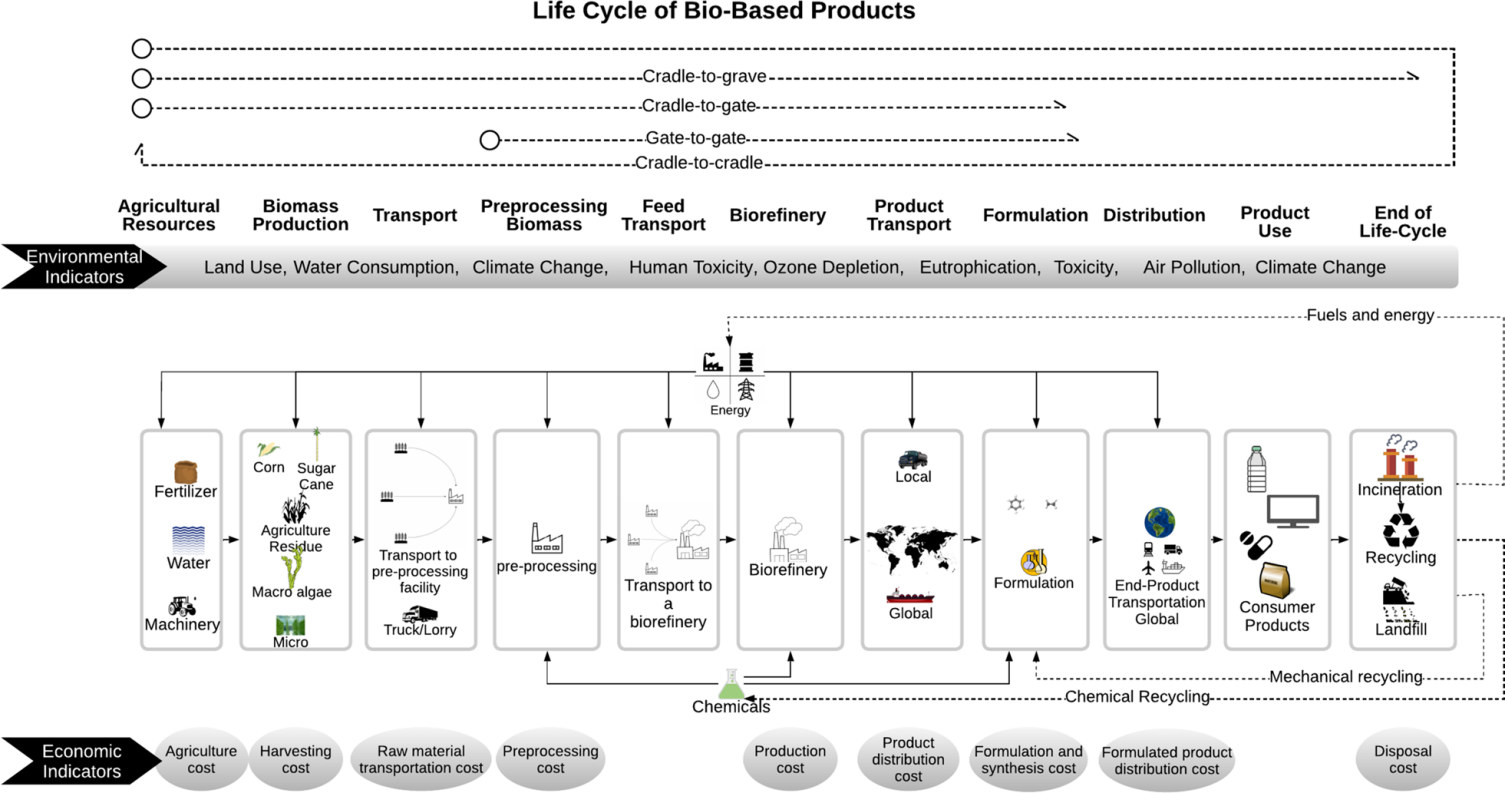
Scheme of bio-based product life cycle stages, economic and environmental trade-offs, and reviewed studies

Based on reviewing the inventories from research contributions in the literature, we employed an illustrative case study to assess and show the trade-offs derived from assessing a product system at high and intermediate maturity levels. Bio-based succinic acid production was chosen, one of the most commercially relevant bio-based building blocks [132]. Figure 3 illustrates a general life cycle of a bio-based product with major interim stages and associated environmental and economic indicators. The detailed calculation used to generate this data set is described in the research data file. Different studies [133, 134] have demonstrated that the biochemical pathways for producing succinic acid are more efficient and environmentally friendly than their petrochemical counterparts [132].

A common functional unit (1 kg of succinic acid) defines the basis for assessing costs and environmental performance. A cradle-to-gate [135] boundary was considered to evaluate impacts over the supply chain. This is an appropriate constraint, since succinic acid is often used as a chemical intermediate for producing other goods in a long value chain. Data on succinic acid production were taken from the studies developed by Moussa et al. [62] (High TRL) and Cok et al. [133] (mid-TRL). More details and inventory data for High and intermediate TRL succinic production are found in the Mendeley research data in Meramo and Sukumara [136] and in the Additional file 1.

Background processes used in the LCA model are based on the Ecoinvent database. ReCiPe End-Point 2016 methodology is used for impact characterization [137]. For the economic assessment, various public and commercial sources were used for estimating feedstock [138], harvesting [138], transportation [139], and production [140] processes. The associated research data display a simplified process diagram of succinic acid production via ammonium sulfate. Sources of background data and the information mentioned above can be found in Meramo and Sukumara [136].

Corn grain was selected as the primary raw material. Depending on the regional biomass availability and planning logistics, biorefinery configuration can be centralized (preprocessing and production at one location) or distributed, with multiple preprocessing locations feeding into a centralized facility. For simplicity, the case study assumes a centralized bioprocessing facility for succinic acid production. The location of the bioprocessing plant is assumed to be in Iowa (United States), considering the high volume of corn grain produced in this region and the presence of a cluster of ethanol production facilities. The region has a matured supply chain supporting the bio-based production of chemicals. Transportation between feed source and biorefinery is about 32 miles [141]. A system expansion approach is implemented to deal with ammonium sulfate as a co-product recommended by ISO 14044 [142]. We assumed that all ammonium sulfate is sold as fertilizer (locally), being a substitute for its petrochemically synthesized equivalent. Since ammonium sulfate is well-established in the chemical market, the system expansion would not affect the current value chain for this substance. The following are the main assumptions for the case study:

- The geographical location of the succinic acid plant in Iowa, USA.
- Allocation of ammonium is handled using system expansion.
- The case study assumes a centralized bioprocessing facility for succinic acid production.
- The potential environmental impacts of packaging are not included.
- The biocatalyst [62] and nutrients used in the fermentation are assumed to be negligible [133].
- Grid electricity production mix (medium voltage).

**Fig. 4:**
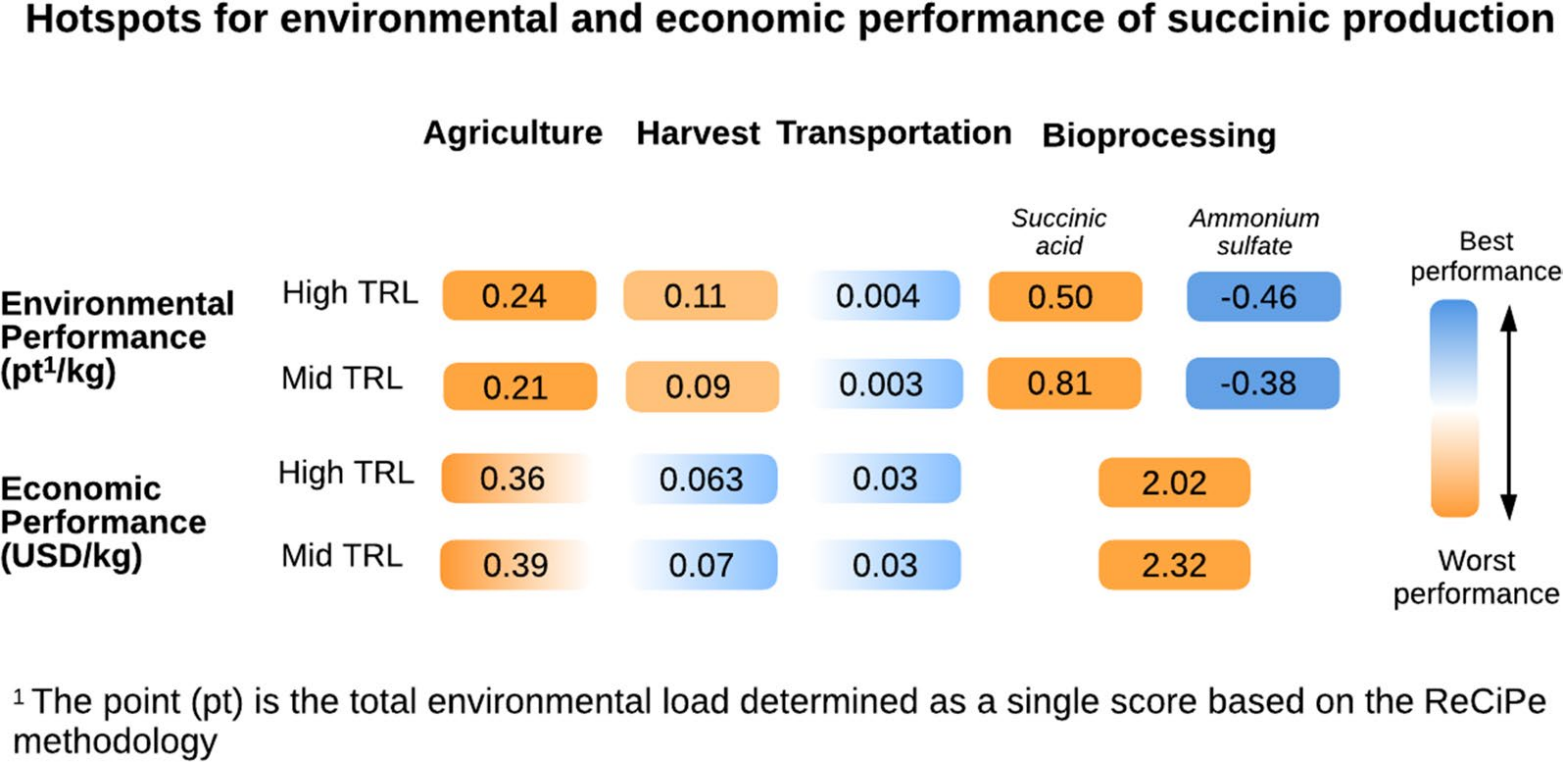
Heat map showing trade-offs between the economic and environmental performance of the succinic acid case study. The numbers in the boxes represent performance or credits (e.g., negatives in ammonium sulfate)

Figure 4 shows a heat map of economic and environmental performance for the succinic acid production case study at different TRLs. The economic and environmental performance present different bottlenecks. The bioprocessing stage is a major driver of the total cost, consistent with the efforts to minimize production, purification, and utility management costs. Conversely, environmental hotspots hint toward the need to improve biomass production performance and succinic acid processing. In both mid- and high-TRL scenarios, transportation is not typically a significant driver of environmental impacts, but most of the impacts come from other stages, such as agriculture or bioprocessing. This is consistent with previous findings that pointed out these stages to be major drivers of impacts [53, 143]. In the case of biomass production, land-use change plays a significant role to the impacts on water consumption and human toxicity, while the biorefinery stage is a major driver of impacts associated with climate change, freshwater eutrophication, and human toxicity. The allocation of ammonium sulfate was avoided by system expansion, and some environmental credits were granted to the process. However, the results might change if one chooses to implement mass or economic allocation [144].

Although the TRLs influence the performance, the relative hotspots prevail in both scenarios. Considering that, the assessment finds the optimization potential at low TRLs; therefore, recognizing a hotspot at an early TRL is recommended. For example, to reduce the relative impact of agriculture, one could consider assessing a different feedstock, keeping track of the impacts, and comparing the performance of proposed alternatives. A persistent challenge is the difficulty of performing comprehensive LCA studies of bio-based products. Very often, both the application and end-of-life cycle phases are dismissed. This is an expected outcome as the dispersed volumes (due to inter-regional trading), transformations, end-use, and disposal scenarios are challenging to be addressed, as encountered in the literature, where most contributions have focused on cradle-to-gate studies [4]. Further adding to the problem, the current coverage of LCA inventories is more limited to upstream components and utilities, so performing cradle-to-grave or even cradle-to-cradle [145] studies can deliver better sustainability performance insights. However, their implementation faces practical technical challenges in tracing and measuring complex formulations which are often not revealed and social challenges, such as tracing consumption of individuals in households, community or region [146, 147].

The challenges are not exclusive of life cycle considerations. Other issues are related to the level of integration connected with technical aspects at the laboratory level. For example, in the early TRL stages, often fermentation media is spiked to reach high conversion rates. However, upscaling this formulation will typically overestimate economic and environmental impacts. Hence, it is not always straightforward to foresee potential impacts at early design phases, but its value is enormous. Accordingly, as the TRL increases, the media components are progressively optimized for a commercially viable operation. Besides, conducting strain design, fermentation, and purification operations using pure sugars such as glucose is a prevailing practice. Cheaper and more environmentally friendly alternatives need to be explored.

## Ways forward to integrate TEA and LCA for bio-based product innovation

Novel ways to address current limitations and successfully develop integrated LCA and TEA methodologies must include practitioners’ perspective, analyzing sustainability from a life cycle thinking. The observed trends point out to invest some efforts in the following aspects.

### Refined monetization factors

Currency and inflation are major factors that highly influence associated uncertainties in adjusting monetization values and involve money change over time [148]. The task involves analyzing such factors considering those variations and their influence in the normalization factors to better deal with the uncertainties. Geographical locations suggest trade-offs between universal monetization procedures and a regional dependence that might change the assessment outcomes. Getting more refined monetization factors is essential to following integration pathways based on combined TEA and LCA indicators.

### Advanced stochastic models

As mentioned in the previous section, lack of data have hindered the appraisal of product use and end-of-life management stages of the entire life cycle. While generating data and corresponding inventory is tedious, opportunities exist for applying stochastic methods to address uncertainties. Recently, Markov chains were used for LCA to analyze clothes reuse in Nordic countries [149]. Scenarios for different uses and disposal methods were assessed. Most LCA studies compared various processing pathways with a similar application phase, including stochastic modeling, which opens a broader way to explore more in cradle-to-grave domains. Variations in the end-of-life cycle phase need to be assessed for each alternative. A cradle-to-grave boundary guarantees thoroughly assessing the environmental impacts of a commodity chemical within the whole life cycle [149]. The described outlook needs to apprehend the economic sustainability dimension to integrate TEA and LCA successfully. In this regard, uncertainties are linked with market issues and the extended life cycle of bio-based products as an intermediate of other products. Special attention must be thought-out, over the linkages of economic and environmental outcomes, for a more accurate assessment.

### Inclusion of computer-aided process design and optimization approaches

Assessing processes for sustainability impacts from the inception to support sustainable innovation in biotechnology is an essential step in R&D. It requires generating data for the low TRL processes, incorporating computer-aided process design [150]. The optimal solution space is explored and bounded using multiobjective optimization algorithms to monitor the trade-offs between economic and environmental indicators. As a decision-making add-on, the optimization module can include a particular feature for a hierarchy-based definition of targets/goals. These goals should come from stakeholders’ expectations, the trends for a specific sector, or competitive markets.

### Machine Learning in sustainability assessment

There is a need to explore more opportunities of using the TEA and LCA indicators to predict sustainability. An integrated model to convert sets of mass, energy, and monetary inputs into outputs in terms of economic (using TEA) and environmental performance (using LCA) facilitates further exploration with computational statistics. In a Machine Learning (ML) context, the input parameters could be categorized as features, which predict their significance to the sustainability scores. Such signals can be fed back to the R&D teams to tune the parameters to optimize the impacts of bioprocess technology, further taking this to the next level. A few cases of such integration have started to appear [151]; nevertheless, its full potential in guiding bioprocess optimization is yet to be explored.

## Conclusions

It is acknowledged that transitioning toward a global sustainable development involves bio-based products with a better environmental and economic performance. Quantitative assessments at early design phases can accelerate the development of bio-based products and guide R&D phases to develop systems directed by maximizing economic and environmental sustainability. Efforts to consistently combine TEA and LCA are described to support decision-making for the R&D stages of bio-based production processes. It is observed that comprehensive, integrated assessments are not usually performed during R&D phases. Performing integrated sustainability assessments would assist stakeholders in foreseeing future product performance in meeting renewable and resource conservation principles and sustainable development goals (SDGs).

Increasing awareness of including sustainability indicators to boost innovation in biotechnology is aligned with global SDGs and moving toward a bioeconomy. In this sense, features of economic and environmental assessments concerning the TRLs, freedom of design, and uncertainty in data must be considered, as illustrated in Fig. 2. The absence of sustainability assessments at early design stages is noteworthy, highlighting the opportunities of performing combined LCA and TEA for bio-based products. Although some efforts have been initiated to integrate LCA and TEA, there is considerable space for science-based methods to accomplish this purpose. In the future, coupling the quantitative sustainability approaches with emerging data-driven learning offers considerable potential to give insights to the R&D process, consequently improving the product's sustainability performance and enhancing the use of renewable resources. In addition, such novel methodological integration could be extended further to address the uncertainties pertaining to data, negating the lack of information in the bio-based product life cycle.

## Supplementary Information

**Additional file 1: Table S1.** Inventory data for succinic acid at high and mid TRL’s. **Table S2.** Sources of background data for the case study. **Table S3.** Damage assessment results for end-point categories. **Table S4.** Normalized damage assessment results for end-point categories. **Table S5.** Economic impact at various stages of high and mid TRL process for producing bio-based succinic acid. **Figure S1.** Simplified process diagram of succinic acid production via ammonium sulfate. **Figure S2.** Damage assessment results. **Figure S3.** Normalized damage assessment results.

## Data Availability

Data for environmental and economic impacts of bio-based production of succinic acid are openly available in [Mendeley data] at [https://doi.org/10.17632/x7fvw5ds5v.1]. These data are also available in the Additional file 1.

## Abbreviations

TEA: Techno-economic Assessment
LCA: Life Cycle Assessment
E/MSY: Extinctions per million species-years
TRLs: Technology Readiness Levels
SWOT: Strengths, Weaknesses, Opportunities, and Threats
R&D: Research and Development
GHG: Greenhouse gas
MCDA: Multicriteria decision analysis
MOO: Multiobjective optimization
KPI: Key Performance Indicators
GREENSCOPE: The Gauging Reaction Effectiveness for the Environmental Sustainability of Chemistries with a Multiobjective Process Evaluator
ROA: Real Options Analysis
ROI: Return on Investment
TOPSIS: The technique for order of preference by similarity to an ideal solution
LCSA: Life Cycle Sustainability Assessment
LCC: Life Cycle Costing
SLCA: Social Life Cycle Assessment
ML: Machine Learning
