# Supplementary material for: Advances and opportunities in integrating economic and environmental performance of renewable products
Source: Biotechnol Biofuels Bioprod. 2022 Dec 22;15:144. doi: 10.1186/s13068-022-02239-2 (PMC9783408; doi:10.1186/s13068-022-02239-2)
Supplement: Supplementary file 1 — Additional file 1: Table S1. Inventory data for succinic acid at high and mid TRL’s. Table S2. Sources of background data for the case study. Table S3. Damage assessment results for end-point categories. Table S4. Normalized damage assessment results for end-point categories. Table S5. Economic impact at various stages of high and mid TRL process for producing bio-based succinic acid. Figure S1. Simplified process diagram of succinic acid production via ammonium sulfate. Figure S2. Damage assessment results. Figure S3. Normalized damage assessment results. [file 13068_2022_2239_MOESM1_ESM.pdf]

Advances and Challenges in integrating sustainability into biotechnology product innovation

Samir Meramo[1], Peter Fantke[2], and Sumesh Sukumara[1,*]

[1]*Sustainable Innovation Office, The Novo Nordisk Foundation Center for Biosustainability, Technical University of Denmark, Kemitorvet 220, 2800 Kgs. Lyngby, Denmark*

[2]*Quantitative Sustainability Assessment, Department of Technology, Management and Economics, Technical University of Denmark, Produktionstorvet 424, 2800 Kgs. Lyngby, Denmark*

*corresponding author: susu@biosustain.dtu.dk

Table S1. Inventory data for succinic acid at high and mid TRL's

Material inputs	High TRL process (Moussa et al., 2016)	Mid TRL process (Cok et al., 2014)
Raw material	1.66 kg	1,83[1]
Dextrose	0.012 kg	N/A[2]
Process water	1.67 kg	32.48 kg
Ultrapure water	25.30 kg	N/A
Liquid ammonium	0.41 kg	0.932
Sulfuric acid	N/A	0.548 kg
Carbon dioxide	N/A	0.165 kg
Utilities		
Total steam	N/A	6.497 kg
Electricity	2.67 kWh	1.995 kWh
Natural gas for steam	14.77 MJ	N/A
Output		
Volatile organic compound	0.002 kg	N/A
Carbon monoxide	0.015 kg	N/A
Carbon dioxide	N/A	1,413 kg
Sulfur dioxide	N/A	7,278 mg
Nitrogen oxide	0.005 kg	0.000743 kg
Particles < 10 µm	0.001 kg	N/A
Ammonia	0.000172 kg	N/A

Sulfuric acid	0.0000806	N/A
Wastewater	0.010 m^3	0.0143 m3
Cell mass	0.820 kg	0.107 kg
Raw material waste	0.395 kg	N/A
Waste sludge	N/A	0.001 kg
Co-products	-	-
Ammonium sulfate	1.49 kg	1.607 kg
Product	-	-
Succinic acid	1 kg	1 kg

[1]Estimated considering a yield of 91% glucose to succinic acid, and a 60% glucose conversion from starch in corn grain(Song and Lee, 2006). [2]Not Apply (N/A).

Table S2. Sources of background data for the case study

**Material inputs**	**Description**
Corn grain	Maize grain
Dextrose	Glucose
Process water	Tap water
Ultrapure water	Water, ultrapure
NH_3	Ammonia, liquid
$Ca(OH)_2$	Lime
HCL	Hydrochloric acid, without water, in 30% solution state
NaOH	Sodium hydroxide, without water, in 50% solution state
H_2SO_4	Sulfuric acid
Utilities	
Electricity	Electricity, medium voltage
Natural gas for steam	Natural gas, burned in gas motor, for storage
Output	
Volatile organic compound	VOC, volatile organic compound
Carbon monoxide	Carbon monoxide, biogenic
Carbon dioxide	Carbon dioxide, biogenic
Sulfur dioxide	Sulfur dioxide
Nitrogen oxide	Nitrogen oxide
Particles < 10 µm	Particulates, < 10 µm
Ammonia	Ammonia
Sulfuric acid	Sulfuric acid
Wastewater	Wastewater/m3

Cell mass	Waste, organic
Raw material waste	Waste, solid
Waste sludge	Treatment of biowaste, municipal incineration
Ammonium sulfate	Ammonium sulfate, as N (0.212 kg N/kg of $(NH4)_2SO_4$)

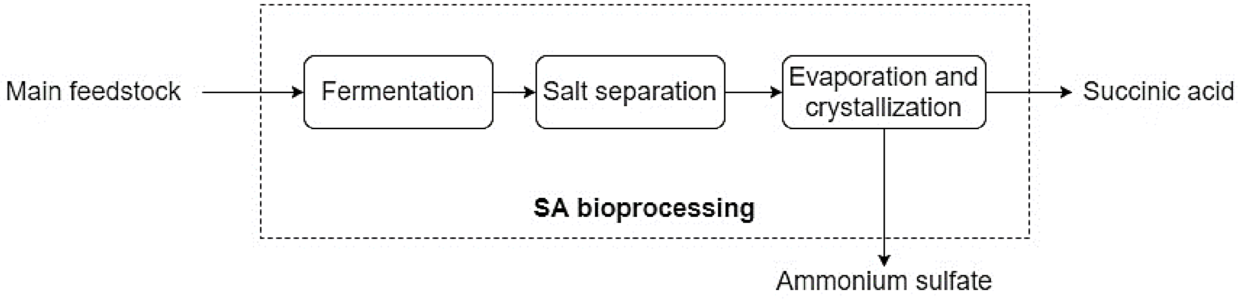

Figure S1. Simplified process diagram of succinic acid production via ammonium sulfate

The LCA was performed for the case study for a succinic acid product based on a cradle-to-gate boundary. SimaPro was employed to perform the environmental assessment. The analysis involves the quantification and comparison of environmental impacts of the case study at different TRLs in order to evaluate the trade-offs regarding the performance considering different levels of maturity. Table S3 and Table S4 summarize the damage assessment results for a succinic acid production. Similarly, Figures S3 and S3 displays the comparison graphically.

Table S3. Damage assessment results for end-point categories

**Category**	**Unit**	**High TRL**	**Low TRL**
Human health	DALY	7.7×10^{-5}	1.9×10^{-4}
Ecosystems	species.yr	8.8×10^{-8}	1.8×10^{-7}
Resources	USD	0.22	0.46

Table S4. Normalized damage assessment results for end-point categories

**Category**	**High TRL**	**Low TRL**
Resources	8.6×10^{-4}	2.1×10^{-3}
Ecosystems	1.0×10^{-4}	2.2×10^{-4}
Human health	7.9×10^{-6}	1.6×10^{-5}

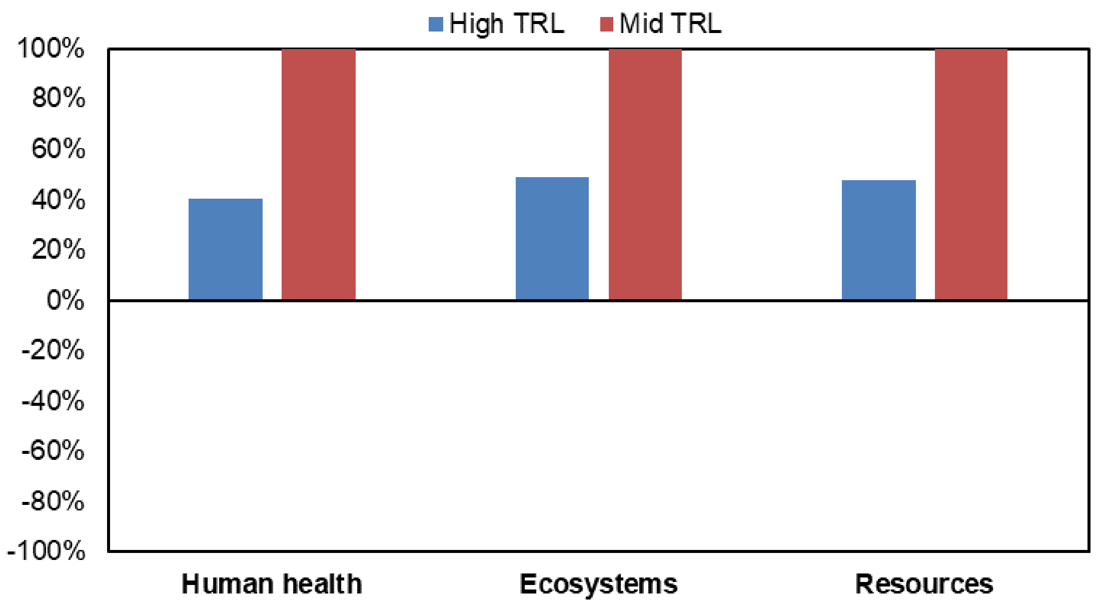

Figure S2. Damage assessment results

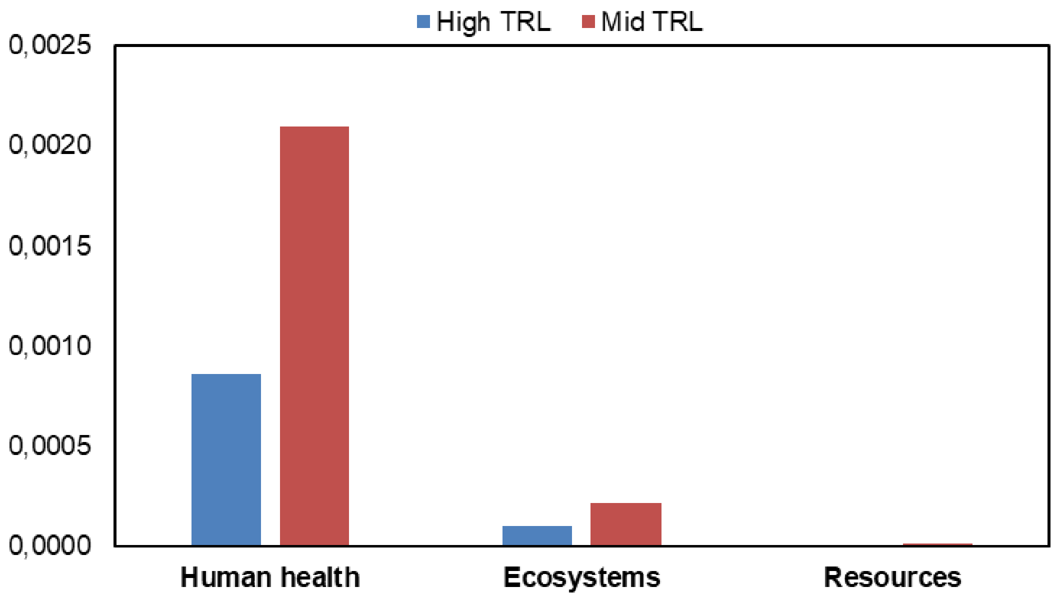

Figure S3. Normalized damage assessment results

Table S5. Economic impact at various stages of high and mid TRL process for producing bio-based succinic acid.

**Process Stages**	**Unit cost for high TRL**	**Unit cost for mid TRL**	**Units**
Cost from agriculture	0,356	0,392	USD/kg of product
Cost from harvest	0,063	0,069	USD/kg of product
Cost from transport	0,027	0,030	USD/kg of product
Production cost	2,018	2,323	USD/kg of product

Supporting information used for estimating cost for the life-cycle stages are obtained from the following sources:

i. Production cost: Commercial database for the cost breakdown of industrial processes. The data used is calculated to produce succinic acid in the USA (IHS Markit, 2017).
ii. Transportation cost: This is based on prevailing costs in the state of Iowa, representing a matured transportation scheme for a biorefinery (Center for Transportation Research and Education, 2010).
iii. Agriculture and Harvest cost: A breakdown of harvest cost in an North American context was obtained, which included detailed share of agriculture and harvesting operations (Manitoba Agriculture and Resource Development Farm Management, 2021; Plastina, 2021).

References

Center for Transportation Research and Education, 2010. Iowa ’ s Renewable Energy and Infrastructure Impacts. Ames.

Cok, B., Tsiropoulos, I., Roes, A., Patel, M., 2014. Succinic acid production derived from carbohydrates: An energy and greenhouse gas assessment of a platform chemical toward a bio-based economy. Biofuels, Bioprod. Biorefining 8, 16–29. https://doi.org/10.1002/bbb.1427

IHS Markit, 2017. Chemical Process Economics Program [WWW Document]. URL

https://ihsmarkit.com/products/chemical-technology-pep-index.html (accessed 8.11.21).

Manitoba Agriculture and Resource Development Farm Management, 2021. 2021 Cost of Production Crops.

Moussa, H.I., Elkamel, A., Young, S.B., 2016. Assessing energy performance of bio-based succinic acid production using LCA. J. Clean. Prod. 139, 761–769. https://doi.org/10.1016/j.jclepro.2016.08.104

Plastina, A., 2021. Estimated costs of crop production in Iowa - 2016. Ame.

Song, H., Lee, S.Y., 2006. Production of succinic acid by bacterial fermentation. Enzyme Microb. Technol. 39, 352–361. https://doi.org/10.1016/j.enzmictec.2005.11.043
